# ‘SWell’ Staff Wellbeing Interventions in Paediatric Critical Care: A Feasibility Study

**DOI:** 10.1111/jep.70092

**Published:** 2025-04-06

**Authors:** Rachel L. Shaw, Amy Fox, Shoshana Gander‐Zaucker, Karen Maher, Sally Crighton

**Affiliations:** ^1^ Institute of Health & Neurodevelopment College of Health & Life Sciences Aston University Birmingham UK; ^2^ Psychology and Human Development Institute of Education, Faculty of Education and Society University College London London UK; ^3^ University of Birmingham Birmingham UK; ^4^ Aston Business School Aston University Birmingham UK; ^5^ NHS England London UK

**Keywords:** critical care, feasibility studies, health personnel, paediatrics, psychological well‐being

## Abstract

**Introduction:**

Paediatric Critical Care (PCC) staff experience high levels of stress, distress and burnout. The objective was to test feasibility of delivering staff wellbeing interventions in UK PCC units.

**Materials and Methods:**

The method was a feasibility study of Staff Wellbeing interventions using standardized psychological measures.

**Study Design and Participants:**

We conducted a multi‐centre feasibility (non‐randomised) study at 14 UK PCC units during 2023. Interdisciplinary PCC staff were recruited through principal investigators (PIs) at each site.

**Data Collection Instruments:**

The primary outcome measure tested was the Short Warwick Edinburgh Mental Well‐Being Scale. Secondary outcome measures tested were: Brief Resilience Scale, Hospital Anxiety and Depression Scale with acceptability and feasibility surveys. All were completed online using Qualtrics.

**Procedures:**

Two ‘SWell’ Staff Wellbeing interventions were tested: Mad‐Sad‐Glad and Wellbeing Images with Appreciative Inquiry. They were low‐intensity, group‐based, structured reflective discussions delivered by PIs. Baseline measures (*t*0) were completed by 596 staff, 264 (43%) completed immediate post‐intervention (*t*1), with 6% and 5% at 3 (*t*2) and 6 (*t*3) months post‐intervention, respectively.

**Results:**

50% (*n* = 14) of UK PCC units delivered 104 interventions to 573 staff demonstrating delivery feasibility.

Wilcoxon signed‐rank tests found that wellbeing scores and depression scores were significantly improved in matched pairs (*t*0, *t*1; *n* = 130). Staff ratings indicated high acceptability and feasibility for incorporating interventions into everyday practice.

**Discussion:**

‘SWell’ interventions are feasible to deliver. Pre/post data collection is possible but significant attrition prohibited long‐term follow‐up. Significant improvements in wellbeing demonstrated appropriateness of outcome measures to detect changes in psychological wellbeing. Further evaluation work is required to determine whether positive changes are sustainable longer‐term.

## 1 Introduction

Paediatric critical care (PCC) is a high‐pressure environment which can be detrimental to psychological health and wellbeing [1, 2]. Improving staff wellbeing has been identified as a priority in future PCC research [3]. The majority of work to date has focused on measuring the prevalence of burnout, post‐traumatic stress and moral distress with little focus on improving staff wellbeing [4, 5, 6]. Although research has attempted to identify coping strategies, there is no evidence demonstrating their effectiveness [7, 8]. Here, we present the first feasibility study of low‐intensity, low‐resource interventions to improve staff wellbeing in PCC units within the UK National Health Service (NHS).

The COVID‐19 pandemic revealed the significance of healthcare staff wellbeing in an unprecedented manner and presented specific challenges worldwide [9, 10, 11]. During this time many UK NHS Trusts introduced schemes to support the wellbeing of their staff, such as mindfulness and yoga sessions. However, these initiatives were considered inaccessible to PCC staff who worked shifts and were described as ‘shiny gimmicky’ offerings that did not meet their needs [12]. A key priority for SWell staff wellbeing interventions then was that they would be accessible to all PCC staff and be deliverable by PCC colleagues in situ (ideally on the unit).

The detailed process of developing the SWell staff wellbeing interventions is reported elsewhere [13]. In short, we reviewed a series of wellbeing initiatives in one PCC unit to determine what Behaviour Change Techniques (BCTs) [14] they employed to identify potential ingredients for a successful intervention in the PCC setting. We gathered empirical evidence across a series of studies and systematic review examining the meanings of wellbeing to PCC staff and what challenged their wellbeing in the workplace [8, 9, 12, 15, 16]. We then used those findings to inform the intervention development process. To develop the SWell interventions, we used the Behaviour Change Wheel to ensure transparency and to ensure the intervention ingredients were theoretically informed [17].

In brief, our previous research highlighted the significance of the team in staff's perceptions of workplace wellbeing [15]. Staff indicated that wellbeing rested upon the coherence of the team in which they worked and them feeling nurtured by their colleagues. This group coherence, or sense of belonging, was identified as fundamental, in line with the self‐determination theory, which identifies belonging or relatedness as essential for people to thrive in work [18]. This led us toward a group‐based intervention which would capitalise on peer or social support as a functional ingredient.

It was clear from our study exploring nurses' experiences during COVID‐19 that competence—or self‐belief—was an essential component of staff wellbeing. Redeployment to adult critical care during COVID‐19 significantly challenged nurses' self‐belief in their competence, which in turn resulted in poor wellbeing [9]. This, too, aligns with the self‐determination theory which includes competence as a prerequisite for staff to thrive in the workplace. It was therefore important for our SWell staff wellbeing interventions to boost staff's confidence in their ability to improve their wellbeing. Following our development of lived experience definitions of wellbeing, we knew that this would require the intervention to offer staff an opportunity to identify what wellbeing meant to them [15]. Through raising their awareness of what wellbeing meant to them, we wanted the intervention to boost their self‐belief in their ability to identity and engage in activities which would improve their workplace wellbeing [13].

For this improvement to be made we predicted that staff would need to monitor their progress, which would both make them aware of their improved wellbeing, but also if the intervention was delivered in a group setting, this would provide social support, which would in turn bolster the sense of belonging so important to workplace wellbeing [15, 18].

The aim of this study was to test the feasibility of delivering ‘SWell’ staff wellbeing interventions [13] in UK PCC units. The objectives are to determine:

1. Whether it is possible to recruit PCC units as intervention sites with dedicated Principal Investigators (PIs) leading intervention delivery.
2. Whether it is possible to deliver training to PIs to enable them to deliver the interventions according to protocol.
3. How feasible it is to recruit staff and for them to attend intervention sessions.
4. How many sessions it is possible to deliver to how many individuals within an 8 month period (determine dose and reach).
5. How feasible it is to gather pre‐ and post‐intervention wellbeing measures.
6. Whether the selected primary and secondary outcome measures are appropriate to detect changes in psychological wellbeing among this population.
7. Whether the SWell interventions are acceptable to those delivering (PIs) and receiving (staff) them.

## 2 Methods

### 2.1 Ethics

Funding was received from Aston University's Proof of Concept fund and NHS England Children's & Young People's Nursing Directorate (C192968).

This study formed part of the wider SWell project. Ethical approval was obtained from the Health Research Authority (HRA) (20/HRA/3817) and Aston University (UREC280719). Individual capability and capacity (C&C) approvals were obtained from Research and Development (R&D) departments at participating NHS Trusts.

The project was led by R.L.S., Health Psychologist with expertise in intervention development and evaluation. A.F. and S.G.Z. were research associates and managed the day‐to‐day management of the project. KM is a psychologist with expertise in wellbeing interventions and contributed to statistical analysis. S.C. is a nurse working as a research fellow with NHS England during the life of this project who provided clinical expertise throughout.

### 2.2 Setting

This study was set in PCC units. We had 17 units volunteer, but 3 withdrew before data collection and intervention delivery began due to workforce pressures, leaving 13 in England and 1 in Scotland, half of the 28 UK PCC units.

Information was gathered by Teams call from PIs at participating units to help describe the settings. Most participating units had psychological support, but few had dedicated time for staff in PCC. All units had a wellbeing lead, but not all these roles were recognised in workload. None of the units had dedicated funding for staff wellbeing and many struggled to engage staff in social activities following the COVID‐19 pandemic. (See File S2 for further information.).

### 2.3 Patient and Public Involvement and Engagement (PPIE)

We set up a national PPIE group consisting of mixed‐discipline clinicians, clinical researchers, and clinical educators with support from the PCC Society (PCCS). The PCCS Wellbeing Specialist Interest Group were involved in the development of the SWell interventions tested here [13]. They supported the research team in establishing key requirements for the interventions, their content and structure, and how they might be delivered. Clinical collaborators in the wider SWell project team and representatives from the PPIE group were invited to SWell study events to support the provision of intervention training and to provide practical expertise when challenges to project delivery were faced.

### 2.4 Participants

Information about the study was circulated to PCC staff via letters to Chief Nurses from NHS England, presentations at meetings of the PCC Operational Delivery Networks (ODNs), and PCCS webinars. Interested staff were requested to contact the research team and subsequently invited to informational webinars and training events. Volunteers were invited to consider taking on the role of PI at their site.

Volunteer PIs (*n* = 14) signed a study agreement form with signed approval from their line manager. PIs were then responsible for working with the Research Integrity Officer at Aston University to obtain C&C approval from their local R&D departments. This included undertaking Good Clinical Practice (GCP), consent taking training, and completing Delegation of Duties logs.

Each PI was responsible for recruiting PCC staff to attend intervention sessions; some recruited colleagues to assist them. They invited staff via email, social media groups, posters on the unit, and word of mouth. All PCC staff across disciplines and levels were eligible to attend intervention sessions. Staff who worked at the site but not in PCC were excluded.

Participant information sheets (PIS) were shared in recruitment emails and social media sites. Clear reference was made to participation being voluntary to avoid coercion. Interested individuals were asked to follow a link to an online PIS and consent form. Before taking part in online questionnaires, participants completed an online consent form providing their informed consent. At the start of each intervention session, staff gave their informed consent via an online PIS and consent form.

### 2.5 Proposed Primary and Secondary Outcome Measures

The proposed primary outcome measure was the short version (7 items) of the Warwick‐Edinburgh Mental Well‐Being Scale (SWEMWBS) [19] which assesses mental wellbeing by asking participants how often they had been ‘feeling optimistic about the future; feeling useful; feeling relaxed; dealing with problems well; thinking clearly; feeling close to other people; able to make up their mind about things’ over the past 2 weeks. Responses range from 1 (none of the time) to 5 (all of the time) on a 5‐point Likert scale. The raw item‐scores are summed and converted to metric total scores using the SWEMWBS conversion table [20]. Scores range from 7 (lowest possible mental wellbeing) to 35 (highest possible mental wellbeing). The wellbeing score is calculated by summing up the score of the items and transforming the scores. The Cronbach's alpha for the scale for the sample was 0.79 showing good reliability.

Selected secondary outcome measures were the Brief Resilience Scale (BRS) [21] and the Hospital Anxiety and Depression Scale (HADS) [22]. The BRS is a 6‐item scale that assesses how well participants bounce back or recover from stress. Each item is rated on a 5‐point scale ranging from 1 = strongly disagree to 5 = strongly agree. Items 1, 3, and 5 are positively worded, and items 2, 4, and 6 are negatively worded. The BRS is scored by reverse coding items 2, 4, and 6 and finding the mean of the six items Scores range from 1 (low resilience) to 5 (high resilience). The BRS scale shows good reliability in the current sample (6 items; *α* = 0.82). HADS has two subscales, each with 7 items measuring anxiety and depression using a 4‐point Likert scale to rate feelings in the past week. The total score is obtained by summing the scores within each subscale. Scores can range from 0 to 21 for anxiety or depression (with mild symptoms scoring 8−10, moderate 11−14, severe 15−21). The Cronbach's *α* for the HADS anxiety and depression subscales also showed good reliability in the sample at 0.82 and 0.81 respectively.

Wellbeing measures were administered at four time points: pre‐intervention (*t*0), immediately after the intervention session (*t*1), and then again at 3 months (*t*2) and 6 months (*t*3) following the session using Qualtrics, a cloud‐based platform for creating and distributing online surveys.

### 2.6 Satisfaction, Acceptability and Feasibility Surveys

In addition, immediately post‐intervention (*t*1), we asked intervention participants to complete a series of questions related to satisfaction, acceptability and feasibility of the SWell interventions. This included Likert scale ratings and open‐ended questions about the intervention sessions in terms of their likes, dislikes, barriers, enablers and suggestions for improvements.

PIs completed a similar survey at the end of the intervention period which also included questions about their experiences of recruiting staff to attend sessions and delivering the intervention sessions.

### 2.7 Data Analysis

The standardised wellbeing measures were scored according to their manuals [19, 21, 22].

Content analysis was used to explore staff's responses to the satisfaction, acceptability and feasibility questions [23]. These responses were coded into categories. Development of the coding frame was iterative meaning that responses were re‐read and compared to the coding frame, and where necessary new categories were created, or existing ones were amended to ensure the meanings conveyed by staff remained consistent across categories [24].

### 2.8 SWell Staff Wellbeing Interventions

Two ‘SWell’ staff wellbeing interventions [13] were tested in this feasibility study. TIDieR (Template for Intervention Description and Replication) checklists are included in File S1. Following the Behaviour Change Wheel analysis of the target behaviour, intervention functions, and assessment of acceptability, practicability, effectiveness, affordability, spill‐over effects, and equity (APEASE), two interventions were built from existing techniques and packaged in a novel way, bespoke for the PCC context. Key requirements were identified in the APEASE assessment and confirmed by PPIE representatives.

Practicability, affordability and equity were especially significant in the design and delivery of interventions because of the requirements of working within the busy, high‐pressure PCC context. That translated to a requirement for the SWell interventions to be low‐intensity to make them deliverable by existing PCC staff trained by us. They would become unsustainable if they were required to be delivered by staff with formal psychological training because of the inequity of psychological support across PCC units and the lack of dedicated funding for wellbeing initiatives. The interventions had to be flexible and deliverable in or near the unit to facilitate attendance and group‐based to make them as efficient as possible. They are described below. As Table 1 illustrates, the two interventions are built from entirely equivalent BCTs. As outlined above, foremost in significance following our exploratory research were social support and self‐belief. We also knew that the interventions would function to provide feedback and monitoring to participants through the process of being engaged in the reflective group discussions.

**Table 1:** Behaviour change techniques and functions in the SWell interventions.

Behaviour change technique (BCT)	BCT description	BCT function	MSG	WI
Social support	To provide support to peers, colleagues, friends, family	To bring staff together to share experiences so they can provide social support for each other	✓	✓
Self‐belief	To boost self‐confidence and one's belief in one's ability to take control of a situation through reflection, practising new skills, and building on previous successes	To build confidence in staff's ability to identify & express their emotions in response to work	✓	✓
Feedback and monitoring	To observe and record what goes well & what could be improved to provide clear feedback enabling staff to monitor the outcomes of their strategies	To encourage staff to monitor what increases their stress, when they experience challenging emotions, what helps boost their wellbeing	✓	✓

Abbreviations: MSG, Mad‐Sad‐Glad; WI, Wellbeing Images.

Unit PIs were given the choice of which intervention they would like to test.

#### 2.8.1 Mad‐Sad‐Glad

The first intervention, Mad‐Sad‐Glad, was identified in our review of existing initiatives [25] and based on a retrospective exercise used in business [26]. A clinical collaborator had been delivering Mad‐Sad‐Glad with newly qualified nurses during the first 6 months of their employment in PCC at one UK unit. They consulted with us to develop a novel bespoke protocol for ‘SWell’ Mad‐Sad‐Glad that was suitable for any member of PCC staff, across disciplines and levels.

Our SWell Mad‐Sad‐Glad intervention involved a structured discussion using prompts to enable staff to identify scenarios at work which had made them feel mad, sad, and glad. Working through each in turn, starting with mad, then sad, and always finishing with glad to facilitate a positive end to the session, probing questions guide staff through the steps of first naming these emotions, attaching them to concrete examples of workplace scenarios, and then identifying whether and how staff currently process those feelings in or outside of work. Feelings are identified individually first, before willing participants share them with the group. A group discussion is then facilitated within a non‐judgemental space, without apportioning blame, where staff are invited to identify how they might recognise these emotions more readily and how workplace scenarios might be changed to improve their experience in the future. (See the TIDieR checklist in File S1 for further details.).

#### 2.8.2 Wellbeing Images (WI)

The second intervention was influenced by our exploratory study which used these techniques [15]. In that study, a range of images were used as stimuli to help PCC staff express what wellbeing meant to them. We conducted individual and group interviews using Appreciative Inquiry [27, 28]. Appreciative Inquiry encourages open‐minded imaginative discussion in a non‐judgemental environment following the pattern of *discovery*: to explore what wellbeing means; *dream*: identifying wishes to improve wellbeing; *design*: determine goals for improving wellbeing; and *destiny*: making it happen by identifying how to overcome challenges and identifying support required. This worked well as a research study and a high proportion of participants reported unprompted how much they had enjoyed the experience of taking part. This led us to repackage the study as an intervention.

In the SWell WI intervention, the process remained largely unchanged except that as an intervention it was always delivered in groups. A systematic review found promising evidence of Appreciative Inquiry as a healthcare intervention technique, especially with organisational change, but concluded that high‐quality evaluations are required to examine its effectiveness for behaviour change and measurable outcomes such as reduced staff turnover and sickness [29]. None of the studies identified were based in PCC and none were designed to improve staff wellbeing. This means the SWell WI intervention using Appreciative Inquiry is the first of its kind to be used in PCC to improve staff wellbeing. (See the TIDieR checklist in File S1 for further details.).

### 2.9 Intervention Training

PIs and colleagues nominated by PIs to help deliver the interventions were trained by the research team and clinical collaborators. Training was delivered in small groups online. It involved an outline of the wider SWell project which informed the development of the SWell interventions described above. The interventions were described including their rationale, intended outcome, techniques and procedure for delivery. Simulated SWell sessions were run involving PIs and colleagues as participants so they could experience it for themselves. Multiple sessions and one‐to‐one support were offered.

## 3 Results

### 3.1 Recruiting PCC Units as Study Sites With Dedicated PI Project Leads

Out of 28 UK PCC units, 14 (*n* = 13 in England; *n* = 1 in Scotland) volunteered to take part. The recruitment strategy had good coverage, achieving participation from 50% of UK PCC units. Each PI was asked to sign an agreement to participate, approved by their PCC unit manager. Once approved, each PI completed GCP and consent‐taking training.

Some PIs recruited colleagues to help delivery interventions. Across the 14 units staff delivering the interventions included: three matrons, four senior sisters, four sisters, one clinical psychologist, one consultant, one staff support nurse, one clinical nurse educator, one quality improvement nurse lead, one individual who was both sister and professional nurse advocate, and one who was both staff support nurse and clinical nurse educator.

PIs provided contextual information relating to their unit's wellbeing provision (see File S2 for more detail). Most had nominated wellbeing leads who undertook those roles voluntarily without remuneration or workload recognition. Some units had received formal training in peer support or stress management, while others had Professional Nurse Advocates who conducted wellbeing supervision sessions and related activities. The provision of psychology services varied, with most being spread across the entire hospital, and some time allocated to PCC staff within a larger portfolio of patient and staff support. Only a few units had funding allocated to wellbeing activities, and where available, it was from Trust charities. Although most units held some wellbeing activities, they had difficulties reinstating them following the COVID‐19 pandemic.

### 3.2 Feasibility of Training Delivery

PIs at each site attended at least one training session and the research team kept in regular contact. Online events were held in November and December 2023 and a face‐to‐face event in February 2024. Unexpectedly, the biggest source of anxiety and requirement for support among PIs was in gaining C&C approvals from R&D departments at their Trusts. The first approval was received at the end of January 2023 and the final approval came in July 2023. It was not possible to begin baseline data collection at sites until R&D C&C had been provided which demanded a staggered start with interventions running from February through to September 2023.

Although questions were asked regarding delivery of interventions, they were largely procedural (e.g., consenting, sharing online survey links). PIs had experience of facilitating group sessions or mentoring and while they had some questions specific to the SWell interventions their competence to deliver them was evident.

### 3.3 Recruitment of Staff to SWell Intervention Sessions

According to recent national data there are 2609.5 whole time equivalent (WTE) nurses across all bands employed in UK PCC units and 332.7 WTE doctors (including medical trainees and consultant paediatric intensivists) [30]. In the participating units there are 1193.4 WTE nurses and 176.8 WTE doctors.^1^ The sample sizes achieved at each stage of this study are expressed as percentages of the numbers employed across all 28 UK PCC units and in the 14 PCC study sites (see Table 2).

**Table 2:** Demographics of participants who completed wellbeing measures.

			Sample as percentage				
Time points	Sample size	Role	28 UK PCC units	14 PCC study sites	Age	Sex	Length of time in profession
Baseline pre‐intervention (*t*0)	596	85% nurses 9% allied health professionals 6% doctors or consultants 2% didn't specify	19.4% — 10.7% —	42.5% — 20.2% —	20−63 years (*M* = 34.4, SD = 9.7)	93% female	9% less than a year 11% 1−2 years 16% 2−5 years 22% 5−10 years 42% over 10 years
Post‐intervention (*t*1)	264	81% nurses 13% allied health professionals 5% doctors or consultants	8.2% — 4.0%	17.9% — 7.5%	20−62 years (*M* = 34.7, SD = 10.1)	92% female	13% less than a year 12% 1−2 years 21% 2−5 years 20% 5−10 years 34% over 10 years
Matched pairs pre‐ (*t*0) and immediately post‐intervention (*t*1)	130	88% nurses 6% allied health professionals 5% doctors or consultants 2% didn't specify	4.4% — 2% —	9.6% — 3.7% —	21−62 years (*M* = 34.32, SD = 9.6)	94% female	10% less than a year 12% 1−2 years 21% 2−5 years 23% 5−10 years 33% over 10 years 2% didn't specify

### 3.4 Intervention Dose and Reach

Twelve units chose to deliver Mad‐Sad‐Glad and two chose WI. During the intervention period, 104 intervention sessions were delivered to 573 staff members with staff attending one session. On average, sessions lasted 60 min with the shortest at approximately 30 min and the longest up to 90 min. Interventions were delivered by PIs with support from colleagues in some cases. Numbers of attendees averaged at 6 with a range from 2 to 10.

### 3.5 Feasibility of Gathering Pre‐ and Post‐Intervention Data

Baseline surveys (*t*0) were released in a staggered manner from January to July 2023 as individual sites gained C&C approvals. Intervention delivery followed this same staggered pattern as C&C approvals were given, beginning in late January and ending across all sites in September 2023. Follow‐up data continued to be collected until the end of February 2024.

The baseline (*t*0) wellbeing measures were completed by 596 PCC interdisciplinary staff members across the 14 units. In total, 573 PCC staff members attended an intervention session. Of those, 264 (43%) completed the immediate post‐intervention (*t*1) wellbeing measures. Attrition rates at 3 (*t*2) and 6 months (*t*3) post‐intervention were high, with only 40 (6%) responses at *t*2 and 29 (5%) at *t*3. It was possible to identify matched pairs of participants who completed wellbeing measures at baseline (*t*0) and immediately post‐intervention (*t*1). This matched pairs sample included 130 PCC staff members whose data were used to perform statistical analyses of wellbeing measures pre‐ and post‐intervention.

Staff characteristics were very similar across samples, which enables us to assume that those who completed wellbeing measures at *t*0 and *t*1 are representative of staff in the 14 participating study sites and across UK PCC units generally. Baseline recruitment was most successful, reaching almost a fifth (19.4%) of UK PCC nurses and approaching half those employed in the 14 study sites (42.5%). Post‐intervention, we achieved a sample of 8.2% PCC nurses employed in UK units and 9.6% employed in our 14 study sites. The matched pairs sample included 4.4% of UK PCC nurses and 9.6% from the 14 study sites.

### 3.6 Appropriateness of Selected Outcome Measures

Analyses were conducted using the selected primary and secondary measures to determine whether they could detect changes in psychological wellbeing following attendance at a SWell intervention. Statistical analyses were undertaken on the matched pair sample (*n* = 130) to enable detection of any change between baseline (*t*0) and immediate post‐intervention (*t*1) measures. Using the sample size of *n* = 130, *α* = 0.01, and effect size *dz* = 0.03 the power analysis via G* Power [31] indicated sufficient power in the analysis (1−β error probability = 0.84).

The data were non‐normally distributed, hence Wilcoxon signed‐rank tests were conducted using SPSS V.29 [32] to investigate the change in scores for wellbeing measures pre‐ and post‐intervention (*t*0−1) for those who took part in an intervention session. The Bonferroni correction was applied to reduce the possibility of getting a statistically significant result (type 1 error) when performing multiple tests. The threshold for statistical significance was defined at the 0.01 level and below.

The Wilcoxon signed‐rank tests showed that significantly more participants indicated a positive difference in their wellbeing scores on the SWEMWBS scale between baseline (*t*0) and post‐intervention (*t*1) than those who indicated a negative difference: *T* = 1729, *z* = 3.24 (2), *p* = 0.001, *r* = −0.20.^2^ The unstandardised effect size (estimated difference between the medians of the populations) is difference (*t*0−*t*1)=0.63, 95% confidence interval (CI) [0.32, 0.93]. Therefore, the wellbeing scores show a significant improvement immediately following the intervention.

For the HADS depression subscale, significantly more participants indicated a negative difference in their depression scores between baseline and post‐intervention than those who indicated a positive difference: *T* = 1538, *z* = −3.45 (2), *p* = 0.001, *r* = −0.21. The unstandardised effect size is difference (*t*0−*t*1) = −0.50, 95% CI [−1.00, −0.50]. Therefore, the depression subscale on the HADS shows a significant reduction following the intervention.

For the HADS anxiety subscale, more participants indicated a negative difference in their anxiety scores between baseline and post‐intervention than those who indicated a positive difference: *T* = 1707, *z* = −2.29 (2), *p* = 0.02, *r* = −0.14. The unstandardised effect size is difference (*t*0−*t*1) = −0.5, 95% CI [−1.0, 0]. This indicated scores on the anxiety subscale decreased following the intervention, however due to our adjusted threshold of *p* ≤ 0.01, the difference is not deemed significant.

There was no significant difference between baseline and post‐intervention ranks on the BRS (*T* = 1933, *z* = −1.14, *p* > 0.05).

In summary, statistical tests showed significant changes in the pre‐ and post‐intervention scores for the SWEMWBS and HADS depression subscale. (See Table 3 for details.) These findings indicate that these measures can detect changes in PCC staff psychological wellbeing. The significant improvements in wellbeing and depression scores can be interpreted as an encouraging sign that the SWell interventions will have a positive impact on staff in the immediate term.

**Table 3:** Wilcoxon signed ranks tests for wellbeing measures pre‐ (*t*0) and post‐intervention (*t*1).

	SWEMWBS	BRS	HADS anxiety	HADS depression
Number of negative ranks (mean rank)	36 (48.04)	44 (43.93)	57 (51.74)	69 (50.89)
Number of positive ranks (mean rank)	68 (55.86)	50 (50.64)	39 (43.77)	31 (49.63)
Tied ranks	26	33	30	27
z‐score	−3.24	−1.14	−2.30	−3.46
Significance value (*p*)	0.001*	0.253	0.021	<0.001*
Effect size (*r*)	−0.20	n/a	n/a	−0.21

* Indicates accepted significance at level < 0.5.

### 3.7 Acceptability of SWell Interventions to Those Delivering and Receiving Them

Analyses here focused on revealing the detail of staff recipients' (*n* = 264, *t*1) perceptions of the SWell interventions and their thoughts relating to their likely implementation within standard practice.

The findings showed a high level of satisfaction with both SWell interventions. In response to how satisfied they were, on a scale of 1−5 (1 = very unsatisfied; 5 = extremely satisfied), ratings were high: 4.5 (SD = 0.6) for Mad‐Sad‐Glad (MSG), 4.6 (SD = 0.5) for WI. In terms of how likely it is that staff would incorporate the SWell interventions into everyday practice, again ratings were high: 4.2 (SD = 0.8) for both MSG and WI.

The aspects of the SWell interventions that staff liked the most included having open and honest discussions, being able to interact with colleagues in a group, realising experiences were shared, and feeling heard. Recipients' dislikes of the interventions included time constraints, being unconvinced about their practical utility (i.e., lack of solutions), and initial discomfort in sharing personal experiences with colleagues (See Table 4 for further detail.).

**Table 4:** Most frequently cited likes and dislikes of the SWell interventions.

Most frequently cited likes			Most frequently cited dislikes		
Category	Illustrative comments	Frequency of responses	Category	Illustrative comments	Frequency of responses
Open and honest discussion	*I enjoyed the sharing of ideas and how people were feeling in an honest and open forum. I think it influences things to change that are an issue for staff wellbeing*. *Being able to talk openly*.	25.7% (47)	Time constraints	*Not enough time to discuss everything*. *Not enough time for everyone to talk*.	21.2% (24)
Group dynamic	*Quality conversations with my colleagues*. *Being able to share ideas with others*.	18% (33)	Lack of solutions	*Issues raised are hard to resolve*. *It acknowledges the issues and challenges that multi‐disciplinary team faces as well as students that will be challenging to fix or are out of the control of the nurses*.	15.9% (18)
Realising shared experiences	*Nice to talk with others and know you aren't feeling alone*. *Felt really good to share experiences as able to relate to each other so felt more united as a team*.	11.5% (21)	Discussing sensitive topics in a group	*You have to feel psychologically safe within the group, to be honest, initially getting used to trusting sharing in a group*.	14.2% (16)
Feeling heard	*Feel like your thoughts are heard and things can be done to help*. *The opportunity to have a voice*.	10.9% (20)	Initial discomfort	*It can be daunting initially, especially in a group where you don't know the people well*.	8.8% (10)
Open and honest discussion	*I enjoyed the sharing of ideas and how people were feeling in an honest and open forum. I think it influences things to change that are an issue for staff wellbeing*. *Being able to talk openly*.	25.7% (47)			
Group dynamic	*Quality conversations with my colleagues*. *Being able to share ideas with others*.	18% (33)			

Enablers included being in a safe non‐judgemental space to discuss sensitive experiences, and the simplicity of the intervention (i.e., its format and the facilitators' skills in creating a supportive and safe space). Barriers mapped closely on to dislikes (See Table 5).

**Table 5:** Most frequently cited enablers and barriers to accessing SWell interventions.

Most frequently cited enablers			Most frequently cited barriers		
Category	Illustrative comments	Frequency of responses	Category	Illustrative comments	Frequency of responses
Group dynamics	*Peers also participating, felt less in the spotlight* *Knowing the colleagues in the chat and feeling as though it is a safe space*	45.2% (75)	Time constraints	*Timings of sessions and if I am here and available*. *Finding the time to do it*.	52.3% (68)
Intervention format	*How easy the concept was. Everything is easy to understand*. *The variety of cards and the fact that it was a visual*	22.9% (38)	Staffing/workload	*Workload, staff shortages, staff opportunities*. *Nurses engaging from home or having time away from the bedside*	13.8% (18)
Facilitator	*Supportive facilitator* *Approachability of the session leader*	12.7% (21)	Discomfort sharing information	*Sometimes get reluctant to express how we feel about certain things. Not able to discuss with other members of the team as easily*.	8.5% (11)
Relating to others' experiences	*Once others expressed their views openly, gave me a bit of confidence to express my opinion as well*	11.4% (19)	Feeling it's not worthwhile	*Unsure how looking at a nice picture can take away the stresses of PICU*. *Nothing much will change*.	8.5% (11)

The biggest challenge faced by Principal Investigators (PIs; *n* = 11 from 10 of 14 units) were workload and time constraints. They reported lack of senior buy‐in and experienced little or no support for the interventions from senior management. When senior management did buy‐into the study, their endorsement made it easier to release staff from the bedside to attend. Most PIs did not receive remuneration (91%) or workload recognition (73%) for delivering the SWell interventions meaning they were undertaken in PIs' own time, unpaid.^3^

Successful methods for improving staff engagement included scheduling intervention sessions on existing study days, positive feedback from colleagues and discussing how changes might be implemented following the intervention sessions. In all, PIs found delivering the SWell interventions very feasible (*n* = 4; 36%) or somewhat feasible (*n* = 7; 64%) which is promising but highlights some issues to be resolved in future implementation and evaluation.

Improvements were suggested by staff attending and delivering the interventions. The most frequent suggestions from attendees related to the intervention format and content (e.g., shorter sessions, longer sessions, larger groups, smaller groups, modifications to the materials for WI) and participation (e.g., including more staff, inviting staff of mixed seniority versus separate groups for differing seniority). Positive comments were made relating to the experience of being involved in the SWell interventions:

Feel it was a worthwhile thing to do and feel the tool could be easily used and replicated. It promoted discussions that were very supportive and made you feel not alone in your worries/stresses.

(SWell recipient)

It's nice to know the culture is being shifted to one that cares for staff as much as their patients.

(SWell recipient)

PIs' suggestions for improvement related to the delivery of the interventions. They wanted more detailed guidance on the theory informing the interventions and delivery techniques. PIs' comments indicated the utility of interventions and echoed the staff's surprise at how insightful they could be:

We have taken some really significant themes from our discussions and been able to implement small but meaningful changes in PICU. Our staff engaged really well, it created a buzz around the unit with members of the team asking if they could be swelled on shift!!… We are keeping it as part of our staff wellbeing package.

(PI)

We have used some of the themes that came up during the intervention sessions and are currently working through them within our shared governance group.

(PI)

This satisfaction and acceptability data will be fundamental in refining the SWell interventions for future longer‐term evaluation and implementation.

## 4 Discussion

This feasibility study has demonstrated that it is feasible to train PCC staff to deliver low‐intensity, low‐resource SWell staff wellbeing interventions. It is feasible to recruit a range of PCC staff to attend them and it is feasible to collect pre‐ and post‐intervention data. It was not possible with the resources available in this study to collect sufficient follow‐up data at 3 and 6 months to enable statistical analyses over time. The primary outcome measure was feasible and showed promising results of a significant positive effect of the SWell interventions. The secondary outcome measures were also feasible to deliver and able to detect change.

The research team were amazed by the success of recruitment to intervention sessions, which was wholly reliant on PIs at each PCC unit undertaking recruitment and consenting either in person on the unit or via internal communications (e.g., emails, closed social media groups). Unfortunately, this was not repeated for follow‐up data collection where there was significant attrition. For staff attending interventions as a one‐off it is not surprising that completing a questionnaire 3 and 6 months later was not a priority for them. Additional on‐site support would be required for more complete data at these follow‐up stages.

Data relating to satisfaction, acceptability, and feasibility illustrates that the SWell interventions—in essence, guided reflective group discussions in a psychologically safe space—were highly attractive to PCC staff. Psychological safety [33] was identified as a necessity and staff recognised the need for those delivering the intervention to have appropriate skills to create that protected, non‐judgemental space in which to share sensitive experiences. With psychological safety as a given, staff benefited from identifying and sharing their sometimes difficult emotions and were bolstered by hearing others experienced similar things.

Provision of psychological support varied greatly across participating UK PCC units, but most had some acknowledgement of staff's psychological support needs in the form of a ‘wellbeing lead’, training staff in stress management, and at very limited sites, provision of funding to host wellbeing activities. Most wellbeing leads—and PIs in this study—were not remunerated for this study, separately from their clinical or other roles. Some roles were not recognised in workloads. This is a representation of a wider lack of buy‐in from senior management in the provision of psychological or wellbeing support for PCC staff. In the sites where PIs did report support from management, they found organising SWell sessions around shifts much more practicable.

The BCTs in the interventions were appropriate to address the target outcome of improved staff wellbeing. The data from wellbeing measures as well as satisfaction and acceptability surveys clearly show that staff benefitted from and valued the social support provided by the SWell interventions. By sharing their similar experiences and emotions, staff improved their self‐belief. The group discussions included feedback and opportunities to monitor feelings and behaviours. Extending the sessions over time and having staff attend more than once throughout the year could further enhance this process and in turn maintain potential improvements in psychological wellbeing.

### 4.1 Strengths and Limitations

To our knowledge, this is the first feasibility study testing staff wellbeing interventions that have been specifically designed for this staff group in the PCC workplace setting. The SWell interventions were designed with practicability, low‐intensity and low‐resource as priorities [13]. This is because the current funding landscape in UK NHS services means there are significantly limited funds available to support staff wellbeing. During the pandemic staff wellbeing hubs were established, but funding has since been withdrawn and services have ceased. A key strength of this study has been prioritising these restrictions and developing interventions which could happen with very small financial commitment. Even if units were to heed advice of remunerating staff time for delivering SWell interventions or were to recognise it in their workload, these costs would be small in comparison to more specialised or high‐intensity psychological services for staff.

This does not mean we do not recognise the need for psychological support for staff. Indeed, we are fully supportive of a model of psychological support which fosters a psychologically‐safe workplace by creating a sense of belonging, encouraging staff to thrive in their day‐to‐day practice, as well as offering increasingly intensive psychological support for those in need or in crisis [16, 34, 35]. By taking this approach, the SWell interventions are fit for purpose and more likely to be adopted into standard practice than more expensive alternatives.

There were some key limitations with the work which future evaluations need to address. The intervention period was not consistent across sites, which meant pre‐ and post‐intervention measures were collected in a staggered fashion across the 14 units. This was caused by significant delays in gaining R&D C&C approvals. This may be due to staff shortages within R&D teams, or it may be the lack of distinction in reviewing protocols for a relatively simple group‐based talking intervention with staff and major Clinical Trials of Investigational Medicinal Products (CTIMP) with an at‐risk patient group. The consequence of this was that units started at different times, meaning interventions were delivered and data were collected at different points in the year (from January to September). During the winter, PCC units often experience peak pressure periods, which arguably differ from those in the summertime (although summer ‘quiet time’ is beginning to disappear in PCC). This could affect staff wellbeing in various ways, but we were unable to identify any potential differences of this kind in our analyses.

While the measures were found to be feasible, on reflection we concluded that the General Health Questionnaire (GHQ12) [36] may be more appropriate for this population. GHQ12 can detect deviations from normal functioning rather than identifying clinical levels of anxiety and depression in a patient population like the HADS does. Furthermore, given the intervention was not targeting resilience—instead, it targeted wellbeing—the team resolved that the BRS was not a required outcome measure for an intervention of this nature.

High attrition rates meant we were unable analyse follow‐up data. In future evaluations, additional resource is required to ensure follow‐up data can be gathered to determine the likelihood of a longer‐term positive impact of the SWell interventions on staff wellbeing. Furthermore, if additional analyses of external factors, such as staff sickness and staff retention, are to be analysed as potential correlations with staff wellbeing, larger longitudinal datasets are required.

While we engaged in PPIE work with PCC staff during the development and delivery of this project, in future work we are keen to involve patients and parents in this process as well, especially since their priorities include improving staff wellbeing [3].

## 5 Conclusion

Low‐intensity, low‐resource staff wellbeing interventions are feasible in PCC. Psychological safety and management endorsement are key requirements. Strengthening PCC staff's group identity—sense of belonging—and facilitating a reciprocity of emotional literacy among colleagues is at the heart of creating a wellbeing‐supportive workplace environment. This study has provided promising means by which we might improve PCC staff wellbeing; we now need to take action to make it happen.

## Author Contributions

Rachel Shaw was chief investigator, responsible for conceptualisation of the project, funding acquisition, managed the project, line‐managed research associates, oversaw data curation and data analysis, and led the writing of the original manuscript draft. Amy Fox was research associate, she led day‐to‐day management of the project during intervention delivery. She conducted all statistical analyses and contributed to event management during the project. She led on writing up the findings sections of the manuscript and has approved the final version. Shoshana Gander‐Zaucker was research associate, she led recruitment and communications with principal investigators, and coordinated data collection. Shoshana contributed to event management during the project. She reviewed and has approved the final version of the manuscript. Karen Maher provided statistical support for the project. She provided support to Amy Fox on the statistical analysis of outcome measures. She reviewed and has approved the final version of the manuscript. Sally Crighton was NHS England's dedicated Nurse Fellow working on the SWell project. She helped coordinate communications with PCC units, gave clinical insight into intervention delivery, and jointly led events held during the life of the project. She reviewed and has approved the final version of the manuscript.

## Conflict of Interests

The authors declare no conflicts of interest.

## Strengths and Limitations

- A key strength of this study is it is the first feasibility study testing staff wellbeing interventions in UK paediatric critical care (PCC) units of which we are aware.
- The intervention development work was guided by empirical evidence and health psychology theory, resulting in a high‐quality and transparent contribution to the evidence base. Central to the success of this multi‐centre study, involving 50% of UK PCC units, was the collaborative approach taken between the central research team and principal investigators at participating units.
- Gaining capacity and capability approvals from Research and Development departments at UK National Health Service Trusts across 14 sites proved challenging and meant significant delays in launching the intervention at some sites (up to 9 months).
- Staggered starts across sites prevented 6‐month follow‐up at three sites and meant maintaining momentum was challenging. Engaging staff to complete follow‐up data requires improvement in future evaluation work.

## Supporting information

Supporting file 1.

Supporting file 2.

## Data Availability

The data that support the findings of this study are available on request from the corresponding author. The data are not publicly available due to privacy or ethical restrictions.

## Endnotes

1 These data are only available for nurses and doctors, reported in the most recent Paediatric Intensive Care Network survey conducted in 2020.

2 The rank mean of one group is compared to the overall rank mean to determine the *z* score. If the groups are evenly distributed, the *z* score will be closer to 0. *T* signifies the sum of ranks of the two that is lower. The *p* value denotes the significance of the test (the probability that the effect due to the intervention rather than chance) and *r* is the odds ratio (the confidence with which we can interpret the effect).

3 This SWell study was funded as a collaborative project which meant PCC units had to cover the cost of intervention delivery themselves. This is different from many externally funded patient research projects which include ‘treatment costs’ for similar activities.
