# Supplementary material for: ‘SWell’ Staff Wellbeing Interventions in Paediatric Critical Care: A Feasibility Study
Source: J Eval Clin Pract. 2025 Apr 6;31(3):e70092. doi: 10.1111/jep.70092 (PMC11973411; doi:10.1111/jep.70092)
Supplement: Supplementary file 2 — Supporting file 2. [file JEP-31-0-s001.docx]

**Supplementary file 2: Wellbeing provision in each PCC unit (anonymized)**

| Unit | Psychology provision | Wellbeing Lead | Peer support activities | Funding | Other wellbeing activities & resources |
| --- | --- | --- | --- | --- | --- |
| 1. | Psychologist, but only small proportion is dedicated to staff support | Wellbeing champions who meet regularly with the psychologists. | N/A | Not aware of any ring-fenced funding for wellbeing | Trust wide support services: Staff Advice Liaison Service (SALS) for counselling, practical help (e.g., financial issues).  Wellbeing resources:1-2-1 drop-in sessions, Psychological debriefs, Teaching on wellbeing, Social events. |
| 2. | Psychologist new in post  Psychological debriefs  1-2-1 sessions | Wellbeing team with a Wellbeing Lead | ‘Seahorse strolls’, paddle boarding. Wellbeing week: newsletter, cycle to work scheme, wellbeing board | Not aware of any ring-fenced funding for wellbeing.  Wellbeing Lead activities undertaken in own time. | Prior to covid: tea & cake sessions |
| 3. | Two Psychologists, no dedicated time for staff yet. | Two consultant Wellbeing Leads in PICU and a nursing Wellbeing Lead. | Peer support team trained in Critical Incident Stress Management | Not aware of any ring-fenced funding for wellbeing.  There is access to funding when needed. | Rolling meeting once a month of the wellbeing and support team.  Initiatives introduced include: cold water tanks, redesigned staff room, removal of clinical information from rest spaces, vending machines of unhealthy snacks replaced with alternative food, ergonomic adjustments to bedspaces.  Social activities: social activities as part of the nursing wellbeing team’s activities. |
| 4. | Psychologist Health & Wellbeing Steering Group. No dedicated time to PICU.  1-2-1 sessions  Debriefs (time-outs) | Wellbeing Lead as part of the Dignity at Work initiative | Health & Wellbeing Champions, Mental Health First-Aiders, Nurse Advocate, Dignity at Work Champion | Not aware of any ring-fenced funding for wellbeing  Staff pay for their own wellbeing activities | Wellbeing champions in PICU organises a monthly walk & cup of tea  Professional Nurse Advocates run drop-in sessions (coffee shop off the unit)  Christmas & summer parties, positivity board, shout-out board |
| 5. | Psychology team in Cardiac care, focuses on patients but do see staff. No psychologist for wider PICU. | Wellbeing department across the hospital dedicated to staff wellbeing, nothing specific for PICU. | Trauma Risk Management training available | Not aware of any ring-fenced funding for wellbeing. | Wellbeing department events: flower arranging, trips, exercise sessions. |
| 6. | Two part-time Psychologists | Five Wellbeing Leads & 12 Wellbeing Champions across the hospital. | One of the wellbeing leads is a trained Professional Nurse Advocate. | Wellbeing Lead activities undertaken in own time. | Professional Nurse Advocate offers: clinical supervision.  Wellbeing activities e.g. breakfast club, Christmas market trips, tea and chat, cook along classes, cocktail classes.  The Trust provides a wellbeing calendar: events for Ramadan, Passover, Diwali, international nurses day, charity work, mental health awareness gardening events.  Mortality meetings monthly where wellbeing needs can be identified. |
| 7. | Psychology support for patients.  Access to 2 external Psychologists.  Debriefs  1-2-1 sessions | Wellbeing Lead in PICU within a core wellbeing team. Wellbeing Lead did the Professional Nurse Advocate training. | Team Emotional Support System training is available in-house.  No trained peer-supporters. | Won charitable funding for 1WTE psychologist for 2 years across the whole Trust ( approx. 15 children’s wards).  Charitable funding paid for the staff wellbeing room.  Wellbeing Lead activities undertaken in own time. | Counsellors (funded externally) and the chaplain are available for staff 1-2-1 sessions.  Star of the month, staff suggestion box, wellbeing room, mentoring for new staff, ‘all about you’ days, |
| 8. | Psychologist at the hospital, not dedicated to PICU or staff. |  | Peer supporters have been available for many years within the nursing team. Clinical supervision is available within the nursing team and for medical trainees. | Charitable fund available for PICU generally, not dedicated to wellbeing. | Wellbeing & resilience project set up in 2017 involving: away days (but expensive), resilience tree, communication tool to boost confidence, clinical supervision, debriefs.  PICU social group: days out, dog walking group. |
| 9. | No Psychologist in PICU. | No Wellbeing Lead but the Professional Nurse Advocate leads many of the wellbeing activities. | Professional Nurse Advocate provides support on PICU for staff. | Not aware of any ring-fenced funding for wellbeing. | Professional Nurse Advocate offers: critical incident stress management debriefs after critical incidents, 1-2-1 debriefs, team debriefs, check-in meetings.  Social activities: coffee mornings & afternoons, monthly activities (e.g., curry night), Christmas party.  Activities have been hard to reinstate after COVID. |
| 10. | 0.2WTE Psychologist on PICU who sees staff and patients. | Wellbeing group involving nurses, consultants, housekeeping staff. | Two Professional Nurse Advocates who aim to provide 1-2-1 sessions with staff (often called away from this work to do clinical shifts when busy). | No pay or financial recognition for wellbeing work. | ‘Good egg award’, wellbeing board, card during COVID to compliment colleagues.  Social events: Christmas party |
| 11. | 0.2TWE Psychologist dedicated to PICU staff.  The psychology team offer:  Consultation  1-2-1 sessions  Post-incident reflective sessions  General reflective sessions  Teaching of wellbeing | Wellbeing Lead in Paediatrics, not specifically PICU.  Informal voluntary wellbeing group running activities in their own time. |  | Not aware of any ring-fenced funding for wellbeing. | Trust initiative offering support for emotional wellbeing, financial wellbeing, how to deal with stress at work, health, smoking cessation, diet & weight loss advice.  Social activities: Christmas parties, Friday social events.  Post-COVID it is difficult to re-engage people in wellbeing activities. |
| 12. | Psychologist on PICU 11 hours/week for patient support, not staff.  Staff support nurse with counselling qualification and Professional Nurse Advocate trained in PICU.  1-2-1 drop-in sessions  Support staff off sick & returning to work  Debriefs | Wellbeing lead on PICU who is the Staff support nurse. | Social events and activities (e.g., food around the world) | Not aware of any ring-fenced funding for wellbeing.  Staff pay for their wellbeing activities. | Wellbeing Lead has created a welcome pack for new members of staff listing wellbeing services available.  Wellbeing lead provides: study days, check-in meetings.  Social activities: climbing group, family fun day, Christmas party |
| 13. | Counsellor on PICU with background in psychology. 1 day/week available to patients & staff on PICU.  Weekly wellbeing meetings  1-2-1 sessions | Two Wellbeing Leads with team of wellbeing advocates who run debriefing meetings when required and signpost external support. |  | No pay or financial recognition for wellbeing work.  Charity donation specifically for social activities and wellbeing. | Team days, but they are difficult to organise with a large staff group & staff living in a large geographical area.  Social activities: pizza night, Christmas party. |
| 14. | Two dedicated psychologists on PICU for patients and staff.  Debriefs offered to staff | Wellbeing lead in nursing. | Team leader (sister/charge nurse) and on starting staff are allocated 1 preceptors (mentors). | No pay or financial recognition for wellbeing work. | Informal wellbeing activities: whatsapp group, secret Santa, staff member of the month, happy events, pictures of long-term patients going home, wellbeing board.  Social activities: Christmas party, new staff drinks, sports day, picnic at the park.  Pre-COVID events included: breakfast after work, mini-golf, drinks. |
